# Assessment of data transformations for model-based clustering of RNA-Seq data

**DOI:** 10.1371/journal.pone.0191758

**Published:** 2018-02-27

**Authors:** Janelle R. Noel-MacDonnell, Joseph Usset, Ellen L. Goode, Brooke L. Fridley

**Affiliations:** 1 Department of Biostatistics, University of Kansas Medical Center, Kansas City, KS, United States of America; 2 Department of Health Services and Outcomes Research, Children’s Mercy Hospital, Kansas City, MO, United States of America; 3 Department of Health Sciences Research, Mayo Clinic, Rochester, MN, United States of America; 4 Department of Biostatistics & Bioinformatics, Moffitt Cancer Center, Tampa, FL, United States of America; University of Nebraska Medical Center, UNITED STATES

## Abstract

Quality control, global biases, normalization, and analysis methods for RNA-Seq data are quite different than those for microarray-based studies. The assumption of normality is reasonable for microarray based gene expression data; however, RNA-Seq data tend to follow an over-dispersed Poisson or negative binomial distribution. Little research has been done to assess how data transformations impact Gaussian model-based clustering with respect to clustering performance and accuracy in estimating the correct number of clusters in RNA-Seq data. In this article, we investigate Gaussian model-based clustering performance and accuracy in estimating the correct number of clusters by applying four data transformations (i.e., naïve, logarithmic, Blom, and variance stabilizing transformation) to simulated RNA-Seq data. To do so, an extensive simulation study was carried out in which the scenarios varied in terms of: how genes were selected to be included in the clustering analyses, size of the clusters, and number of clusters. Following the application of the different transformations to the simulated data, Gaussian model-based clustering was carried out. To assess clustering performance for each of the data transformations, the adjusted rand index, clustering error rate, and concordance index were utilized. As expected, our results showed that clustering performance was gained in scenarios where data transformations were applied to make the data appear “more” Gaussian in distribution.

## Introduction

The analysis of RNA-Seq data comes with some different and additional challenges, as compared to microarray based data. In contrast to microarray based mRNA data, in which relative mRNA is measured for pre-defined probe sets using fluorescence, RNA-Seq experiments measure the mRNA gene expression levels from the total number of reads that map to the exonic regions of the genome. Thus, the quality control measures, global biases, normalization techniques, and analysis methods vary between the two mRNA data types. While statistical analysis methods are capable of handling microarray and RNA-Seq data similarly, analysis methods used for microarray based mRNA studies cannot be implemented in the same fashion to sequence based mRNA studies due to unique data properties. In particular, microarray mRNA data can be assumed to follow a continuous distribution (e.g., Normal/Gaussian distributions), whereas, sequencing based mRNA data follow a discrete distribution (e.g., over-dispersed Poisson/Negative Binomial distribution).

A common goal of RNA-Seq studies is to determine subtypes or clusters of individuals based on their transcriptomic profiles. Cluster analysis has the ability to show genes with common roles and functions in the cellular process cluster together, determine prognostic clusters and clusters based on some marker of health status, and identify subtypes of invasive cancers [1–6]. The challenge for clustering analysis lies in utilization of the most appropriate clustering method, and in turn coming up with both the “correct” number of clusters and assignment of samples to clusters [7]. One particular type of clustering method used often is model-based clustering, which models the data as coming from a distribution that is mixture of two or more components. Often, model based clustering uses a mixture of Gaussian distributions, as implemented in the R package *mclust* (https://cran.r-project.org/web/packages/mclust/index.html) [8, 9].

Over the past two decades, several researchers have assessed clustering methods for mRNA data generated by microarray [1–3, 10–14]. However, little research has been done to assess how cluster methods perform in the analysis of RNA-Seq data and if transformation of the data can improve the performance. RNA-Seq data have three problematic properties when it comes to statistical analysis, including clustering analysis: (1) a skewed distribution, (2) variability among the read counts for individual genes, and (3) likelihood of extreme values [15]. The skewness of the distribution can be addressed by using a data transformation. Application of data transformations to meet assumptions and make analysis methodologies more efficient are very popular, and have been used in RNA-Seq studies. Current literature contains three closely related studies that have looked at performance of clustering methods for sequence data: the first one investigated clustering of sequencing data using a Poisson log-linear model [16]; the second looked at consistency of results from differential expression and clustering analyses between the two technologies for assessing mRNA (microarray and sequencing) using a variety of statistical methods [17]; and the last study provided a model-based clustering framework for determining groups or sets of differentially expressed genes using RNA-Seq data [18]. In this paper, we set out to evaluate how the commonly used Gaussian model-based clustering method performs when applied to RNA-Seq data after a variety of data transformations were applied, with the ultimate goal of clustering subjects/individuals in to distinct molecular subgroups.

## Materials and methods

### Mayo Clinic ovarian cancer RNA-Seq study

Motivation for this study came from an ongoing ovarian cancer RNA-Seq gene expression study which seeks to examine relationships between the different invasive ovarian cancer histologies and variation in the transcriptome [19]. Additional information on ovarian cancer cases included in this study and the experimental methods can be found in the manuscript by Earp et al. [19]. Fresh frozen tumors were obtained from women with ovarian cancer seen at the Mayo Clinic in Rochester, MN. RNA-Seq was performed in four batches, with only batch 1 data used in this study. Briefly, for batch 1, 1000ng of RNA was processed using Illumina’s TruSeq Stranded Total RNA Library Prep Kit with sequencing completed at BGI Americas with paired end (PE) 100-nucleotide (nt) reads. Primary analysis and de-multiplexing was performed using Illumina’s *CASAVA* software, followed by alignment using *TopHat2* [20] and abundance estimation at the gene level using *RSEM* [21]. In order to investigate potential data transformations that improve clustering performance, “real-life” data parameters were acquired from the 55 high-grade serous histology tumor samples in batch 1 which were selected due to their commonness, aggressive nature, and uncertainty surrounding the number of potential subtypes present within this histology—ranging from two to five subtypes [4–6, 22, 23]. Additionally, we applied the model-based clustering to the RNA-Seq data collected on the 55 serous histology tumor samples following various transformations of the data. Data used in the clustering can be found in S1 Table (data on the top 100 MAD genes) and S2 Table (data on randomly selected 100 genes).

### Simulation study

**Fig 1:**
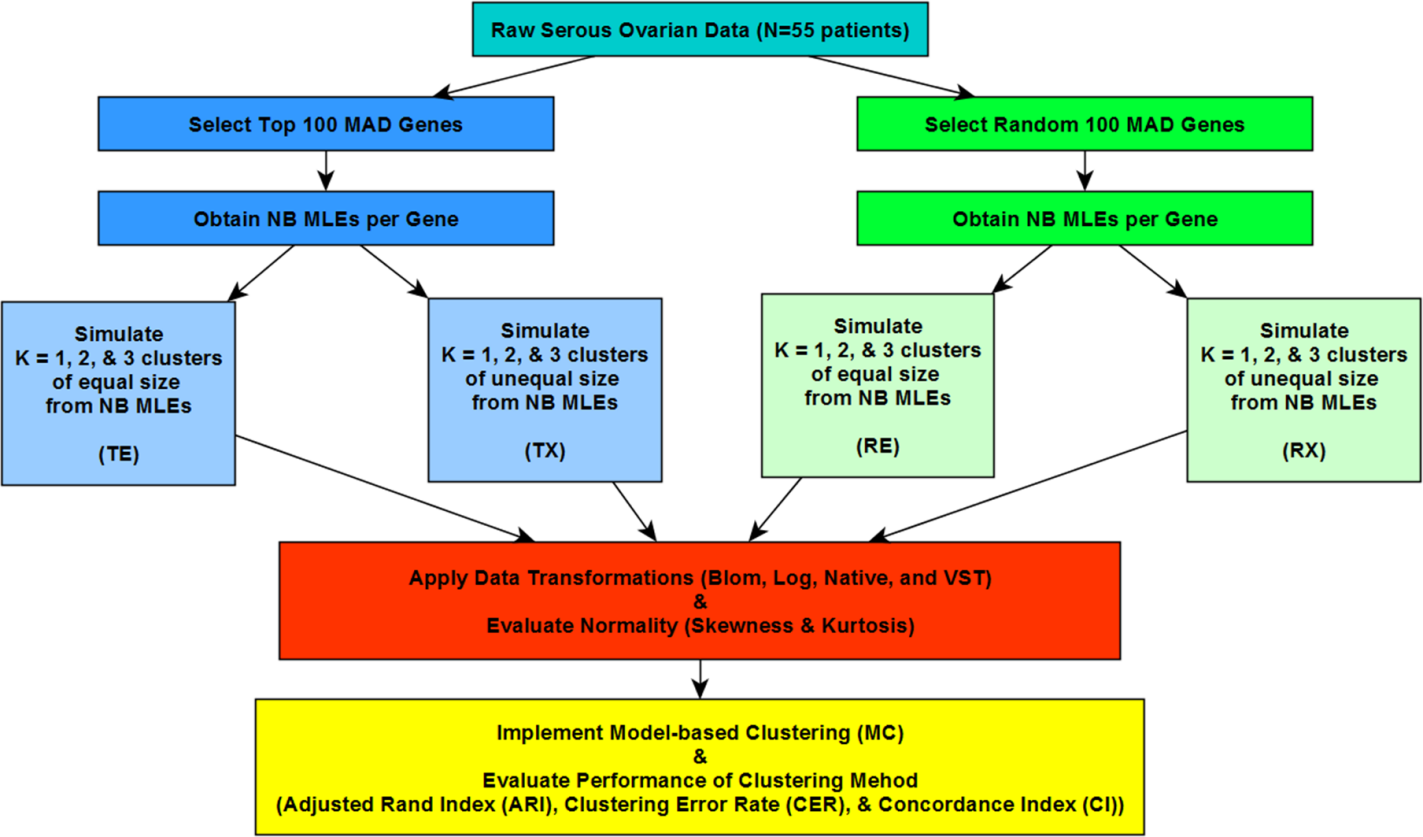
Simulation schematic. Negative binomial (NB) parameters were obtained from 100 top genes and 100 randomly selected genes based upon Median Absolute Deviation (MAD) of expression values taken from ovarian cancer RNA-Seq tumors (N = 55 patients). Data were then simulated to reflect varying cluster sizes, equal and unequal, for K = 1 (i.e., no clusters), 2, and 3 clusters using the NB parameters. One hundred datasets were simulated for four parent dataset categories which reflected gene selection and cluster size (1) Top 100 genes with equal cluster sizes (TE); 2) Top 100 genes with unequal cluster sizes (TX); 3) Random 100 genes with equal cluster Sizes (RE); and 4) Random 100 genes with unequal cluster sizes (RX)). Data transformations and model-based clustering were applied to all datasets and evaluated according to normality measures and clustering evaluation metrics.

To address the aims of this study, an extensive simulation study focused on sample-based clustering was conducted, as outlined in Fig 1. The factors varied in the simulation study included: strategy for how genes were selected to be included in the clustering analyses (top 100 genes according to their median absolute deviation (MAD), or random sample of a 100 genes); size of the clusters (equal cluster sizes or extremely unequal cluster sizes); and number of clusters K = 1 (i.e., no clustering), 2, and 3 clusters. The simulated datasets can be organized into four parent categories reflecting gene selection and size of clusters: top 100 MAD genes with equal cluster sizes (TE); random 100 genes with equal cluster sizes (RE); top 100 MAD genes with unequal cluster sizes (TX); and random 100 genes with unequal cluster sizes (RX). For parent categories with equal cluster sizes, clusters had 28 and 27 samples for K = 2 clusters, and 18 samples for two clusters with one cluster containing 19 samples for K = 3 clusters. Conversely, unequal cluster scenarios had 5 samples in cluster one (*c*_1_) and 50 samples in cluster two (*c*_2_) in K = 2 clusters; along with, 5, 17, and 33 samples for *c*_1_, *c*_2_, and *c*_3_ (i.e., cluster three), correspondingly in K = 3 clusters.

One hundred datasets were simulated for each of the four parent categories prior, followed by applying one of 4 transformations. The four data transformations utilized were: naïve (no transformation); logarithmic base 2 (Log); Blom [24]; and variance stabilizing transformation (VST) [25]. These transformed datasets were then evaluated for normality by looking at measures of skewness and kurtosis. Next, Gaussian model-based clustering (MC) using *mclust* [8] was carried out on all simulated datasets and assessed for clustering performance through use of the adjusted Rand Index (ARI) [26], Clustering Error Rate (CER) [16], and Concordance Index (CI or C-Index) [27] (Fig 1). All analysis for this study were conducted in R statistical software [28].

Before assessing our data transformations and model-based clustering performance, great consideration was given to the way in which the data are simulated to ensure that the simulated data would be similar to what would be found in a real RNA-Seq experiment. Often researchers have simulated RNA-Seq count data from a negative binomial distribution. The negative binomial distribution allows for two distributional parameters to be controlled—the mean and shape—allowing researchers to model the over-dispersion which typically exists in sequencing data. In this study we simulated Negative Binomial data using parameter estimates based on RNA-Seq data from an ovarian cancer study in the hope that our simulated data will better resemble that of “real-life”.

#### Feature selection

The ovarian cancer data set contains gene abundance estimates for *G* = 63,152 Ensembl gene IDs on *N* = 55 serous tumor participants. Let X^*^ be the *G* by *N* matrix where x^*^_gi_ is the expression level count for the *g*^th^ gene (*g* = 1,…, *G*) and the *i*^th^ sample (*i* = 1,…, *N*). As clustering if often done on a subset of the genes, we looked at two methods for determining the genes to be included in the clustering: 1) selecting 100 of the top most variable genes (most common practice in selecting genes for clustering), and 2) selecting a random sample of 100 genes. The top 100 most variable genes were selected by calculating each gene’s median absolute deviation (MAD) resulting in a reduced matrix of 100 genes measured on the *N* samples, denoted by ***X***_*T*_. Similarly, a dataset that contains 100 randomly selected genes, where prior to obtaining a random sample of 100 genes, we filtered out the lower 50% MAD genes (i.e., removed the non-expressed genes). Then, from the remaining genes we randomly selected 100 genes and stored in a matrix denoted ***X***_*R*_.

#### Maximum likelihood estimators for the negative binomial simulation parameters

Vector Generalized Linear Models (VGLMs) are an inclusive class of models of various multivariate response types that are highly generalizable [29, 30]. VGLMs are models of the form

$$f(y|x;B)=h(y,\eta_{1},\ldots ,\eta_{M},\varphi )$$

for some known function *h*(∙), where ***B*** = (*β*_1_*β*_2_ … *β*_*M*_) is *p x M*, *φ* is an optional scaling parameter, and $\eta_{j}=\beta_{j}^{\prime }x=\beta_{(j)1}x_{1}+\ldots +\beta_{(j)g}x_{g}$ is the *j*th linear predictor [29]. Once the form of the model is established, the log-likelihood function can be obtained and Maximum Likelihood Estimates (MLEs) can be found for the parameters in the parent distribution through Iteratively Reweighted Least Squares (IRLS) using either the Newton-Raphson or Fisher-scoring algorithm[29, 31]. To obtain data that reflect that of “real-life”, we utilized VGLMs to obtain MLEs from fitted negative binomial models for each gene using VGLMs using the R package *VGAM*. The subsequent MLEs were used in the simulation of the RNA-Seq data where simulated data for gene *g* and sample *i* was simulated from $x_{gi}∼NB({\hat{\mu }}_{g},{\hat{k}}_{g})$, where ${\hat{\mu }}_{g}$ is the MLE of the mean and ${\hat{k}}_{g}$ is the MLE for the dispersion parameter.

**Fig 2:**
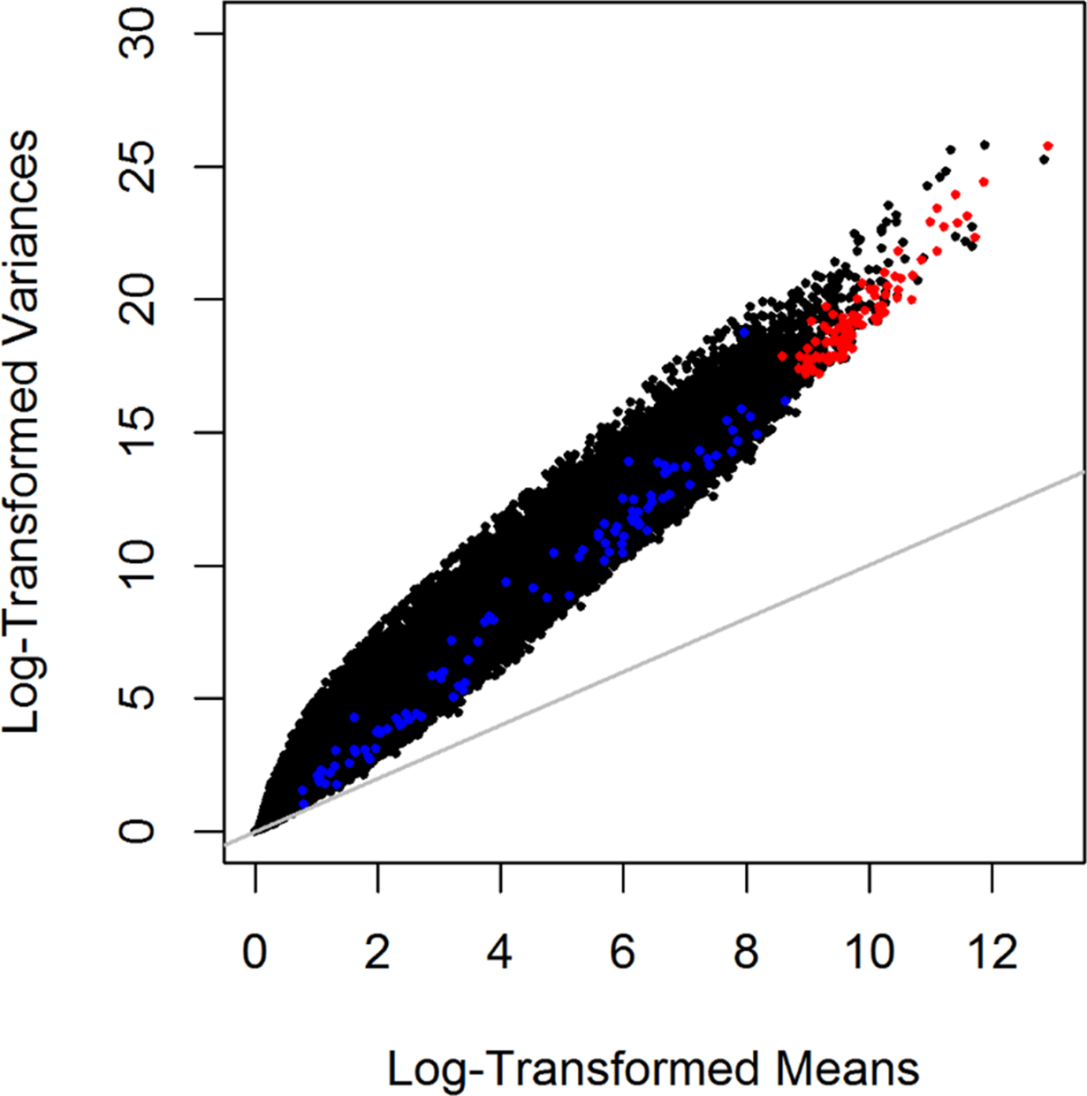
Comparison of raw serous expression counts verses simulated data. Log-transformed mean and log-transformed variances are plotted for comparison of raw serous expression count data and simulated data scenarios for a single dataset. Data points depicted in red are representative of data simulated using Negative Binomial (NB) parameters from the top 100 genes; and similarly in blue, simulated data from 100 randomly selected genes based upon Median Absolute Deviation (MAD) of expression values taken from ovarian cancer RNA-Seq samples (N = 55 tumors).

In order to simulate data that that contained “clusters”, we incorporated effect size shifts to ${\hat{\mu }}_{g}$ and ${\hat{k}}_{g}$ to a proportion of genes which would represent genes that were up-expressed in this cluster group. Fig 2 shows comparison of one simulated dataset to the real RNA-Seq data on the ovarian cancer tumors (represented in black). The data points depicted in red represent the data simulated for a scenario in which the 100 most variable genes based on MAD were simulated, while the points in blue represent data simulated for the scenario involving 100 randomly selected genes. To achieve clustering of samples, we set 10% of the genes in any dataset to be up-expressed for *K* = 2. Likewise, for *K* = 3 a step progression in the percentage of genes that were up-expressed was implemented—10% for *c*_2_ and 20% for *c*_3_. For consistency, the 10% of up-expressed genes in *c*_2_ remained the same in the simulations for K = 2 and K = 3. Based on an empirical study, the effect size shifts for the mean and dispersion parameters were set to $\Delta_{{\hat{\mu }}_{1}}=\exp\left(3.375\right),\Delta_{{\hat{\mu }}_{2}}=\exp\left(5.5\right),\Delta_{{\hat{k}}_{1}}=1.01$, and $\Delta_{{\hat{k}}_{2}}=1.03$.

### Model-based clustering and performance evaluation metrics

In model-based clustering (MC) the data is assumed to be from some finite mixture of probability distributions (i.e., a mixture of Gaussian models). Moreover, the likelihood of the mixture model can be written as $L(\theta_{1},\ldots ,\theta_{K}|X)=\prod_{i=1}^{N}\sum_{c=1}^{K}\tau_{c}f_{c}(x_{i}|\theta_{c})$, where *K* is the number of clusters or components in the data, ***x***_***i***_ are the independent multivariate observations, *f*_*c*_ is the density of some multivariate normal distribution distributional model with mean of *μ*_*c*_ and covariance matrix ∑_*c*_, *θ*_*c*_ are the parameters for the *c*^th^ component which can be thought of as the k^th^ cluster, and *τ*_*c*_ is the probability that an observation belongs to the *c*th component; *τ*_*c*_ has two restrictions: *τ*_*c*_ ≥ 0 and $\sum_{c=1}^{K}\tau_{c}=1$. Utilizing the *mclust* package in R, we are seamlessly able to implement this model-based clustering approach as proposed by Farley and Raftery in 2002 [8, 32]. As we wanted to optimize clustering performance in every method that we used in the simulation, we used the *mclustBIC()* function, which determines the optimal value for the number of clusters *K*.

To summarize and compare the performance of model-based clustering for each of the data transformations, the following three evaluation criteria were used: Adjusted Rand Index (ARI) [26], Classification Error Rate (CER) [16], and the Concordance Index (CI or C-Index) [27]. The ARI ranges in value between 0 and 1 and is computed as a measure of cluster similarity [17, 26]. Values near 0 represent a lack of samples clustering to their “true” cluster; whereas, 1 indicates that samples cluster perfectly. The CER is similar to the ARI; however, it is essentially the complementary calculation without the adjustment. Additionally, the CER is can be computed as 1 minus the Rand index[33]. Lastly, the CI the probability that Sample *j* will cluster to *c*_1_ if the sample was initially from *c*_1_. A CI value equal to 0.5 means that the probability of predicting the correct cluster assignment is no better than that of random chance or that there is no predictive ability. Values of CI that are closer to 1 indicate high predictive ability for objects to be clustered perfectly [27].

## Results

### Normality assessment of data transformations

To compare the data transformations, measures of skewness and kurtosis were evaluated. All data transformation which numerically changed the data (i.e., Blom, Log, and VST) had skewness values more similar to that of a Gaussian distribution as compared to the naïve transformation (Table 1). However, the kurtosis values corresponding to the more normal skewness values were platykurtic (kurtosis value < 3). Skewness values closest to 0 for the RE and RX parent scenarios came from the Blom transformation and for the TE and TX scenarios from the VST transformation. Values for both skewness and kurtosis remained the same when K = 1 across all transformations and parent scenarios, implying that method of data selection did not play a role in determining normality. For simulated clusters of K = 2 or K = 3, it is likely that the combination of varied cluster sizes and the effect shifts implemented in the NB distribution to form clusters played a role in the differences in normality between parent categories.

**Table 1:** Mean skewness and kurtosis for simulated data scenarios.

Transformation	No. Clusters	Parent Category							
		TE		RE		TX		RX	
		Sk	Kt	Sk	Kt	Sk	Kt	Sk	Kt
Naïve	K = 1	1.40 (0.005)	2.45 (0.029)	1.48 (0.006)	2.78 (0.035)	1.40 (0.005)	2.45 (0.029)	1.48 (0.006)	2.78 (0.035)
	K = 2	1.46 (0.006)	2.67 (0.035)	1.52 (0.005)	2.88 (0.031)	1.40 (0.006)	2.43 (0.033)	1.47 (0.006)	2.75 (0.032)
	K = 3	1.70 (0.005)	3.98 (0.026)	1.60 (0.005)	3.15 (0.031)	1.47 (0.005)	2.57 (0.027)	1.56 (0.005)	2.94 (0.029)
Blom	K = 1	-0.29 (0.002)	-0.43 (0.005)	-0.37 (0.003)	0.16 (0.011)	-0.29 (0.002)	-0.43 (0.005)	-0.37 (0.003)	0.16 (0.011)
	K = 2	-0.31 (0.002)	-0.46 (0.005)	-0.34 (0.003)	0.13 (0.011)	-0.47 (0.002)	-0.17 (0.006)	-0.52 (0.004)	0.56 (0.013)
	K = 3	-0.31 (0.003)	-0.28 (0.004)	-0.29 (0.004)	0.24 (0.011)	-0.52 (0.003)	0.31 (0.007)	-0.39 (0.004)	0.55 (0.013)
Log	K = 1	-0.77 (0.004)	0.75 (0.014)	-0.43 (0.003)	0.01 (0.010)	-0.77 (0.004)	0.75 (0.014)	-0.43 (0.003)	0.01 (0.010)
	K = 2	-0.71 (0.004)	0.60 (0.015)	-0.38 (0.003)	-0.13 (0.009)	-0.80 (0.004)	0.77 (0.016)	-0.51 (0.003)	0.14 (0.010)
	K = 3	-0.67 (0.004)	0.42 (0.013)	-0.40 (0.003)	-0.18 (0.008)	-0.80 (0.004)	0.70 (0.013)	-0.49 (0.003)	-0.002 (0.009)
VST	K = 1	-0.18 (0.037)	-0.37 (0.121)	-0.50 (0.007)	0.19 (0.014)	-0.18 (0.037)	-0.37 (0.121)	-0.50 (0.007)	0.19 (0.014)
	K = 2	-0.58 (0.063)	0.62 (0.233)	-0.52 (0.007)	0.10 (0.013)	-0.72 (0.071)	1.22 (0.300)	-0.61 (0.006)	0.34 (0.014)
	K = 3	-0.48 (0.047)	0.04 (0.164)	-0.57 (0.005)	0.20 (0.013)	-0.56 (0.036)	0.23 (0.114)	-0.71 (0.004)	0.42 (0.012)

Sk, mean (standard error) skewness; Kt, mean (standard error) kurtosis

### Evaluation of model-based clustering by data transformation

**Fig 3:**
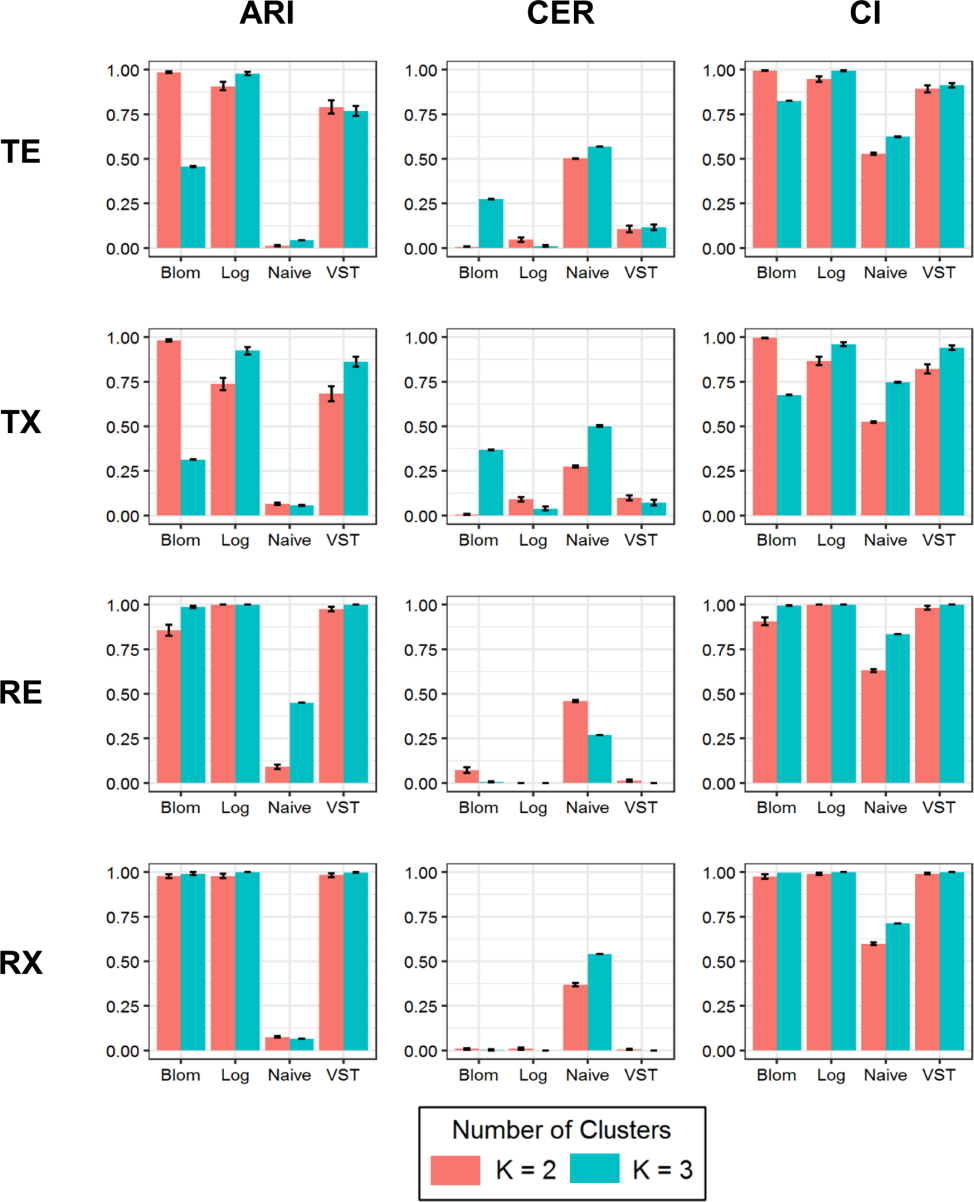
Comparison of model-based clustering evaluation criteria. Mean Adjusted Rand Index (ARI), Clustering Error Rate (CER), and Concordance Index (CI) are plotted for each of the four parent dataset categories for K = 2 clusters (in coral) and K = 3 clusters (in teal).

Clustering method performance was measured for all 96 simulation scenarios. However, the assessment of K = 1 scenarios are not presented in either of the summary tables as there were not multiple clusters to compare sample assignments. For K = 2 and K = 3 all data transformations, with the exception of the naïve transformation, had performance values that were better than random chance with mean CI values are greater than 0.5 (Fig 3). When looking at the ARI and CER it is apparent that differences do exists between pairings of data transformation and type of clustering method used. Notably, model-based clustering did not perform well in regards to selecting the correct clustering assignment when the Blom transformation is used with data that are highly variable prior to the transformation, that is for those data that represent the top 100 MAD genes (TE and TX) for K = 3. The best overall performance was observed when the log transformation was applied to datasets simulated with K = 3. Furthermore, in general the parent dataset category does not appear to have an effect on performance, with the exception of the Blom transformation applied to TE and TX parent categories. The Blom, Log, and VST transformations have similar results across the evaluation metrics for data that were simulated from the selected random 100 MAD genes.

### Clustering of ovarian cancer study

**Fig 4:**
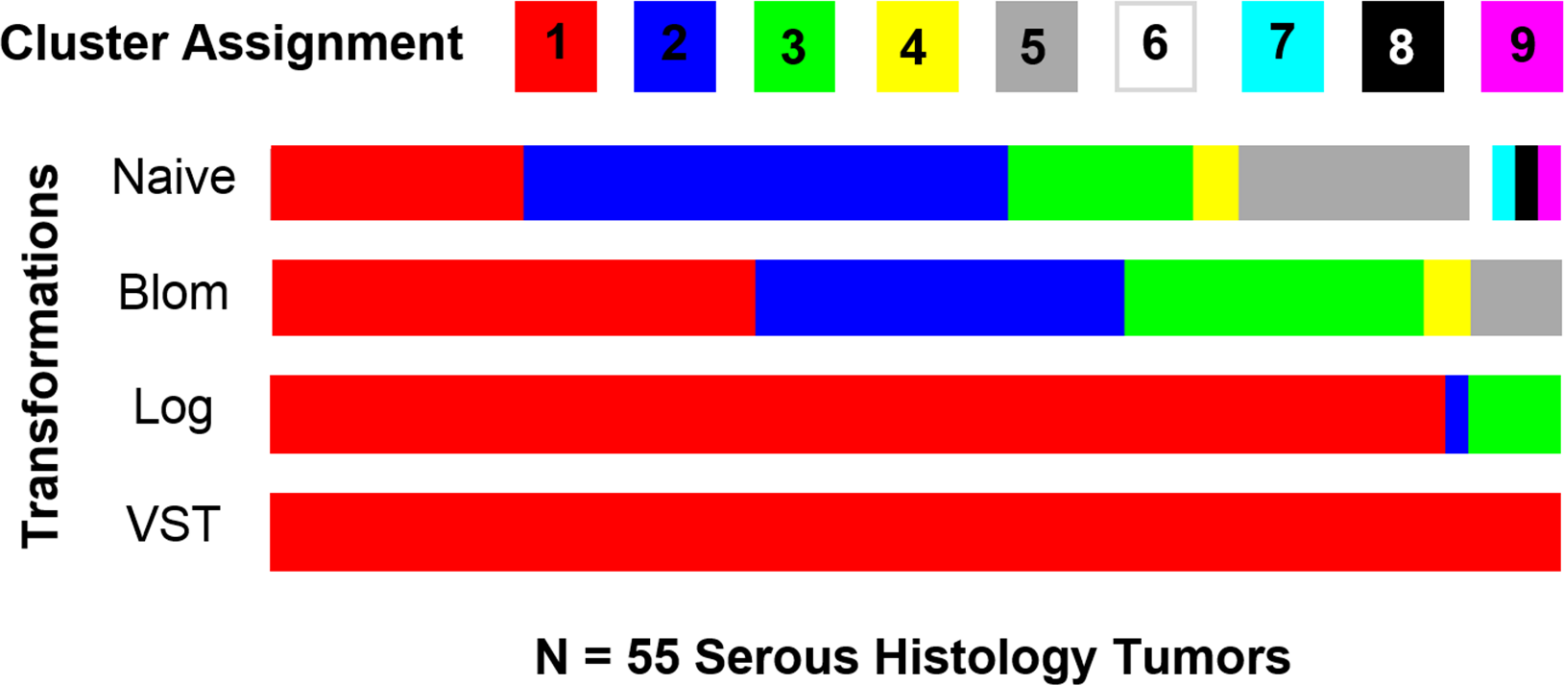
Model-based clustering assignments of 55 serous histology tumors. Model-based clustering was carried out on the raw serous ovarian data following data transformation. Clustering assignments for each of the tumor samples are denoted using the various colors. The width of a specific cluster assignment’s color in a given transformation is representative of the amount of samples clustering together.

Model-based clustering was also applied to the raw and data transformed RNA-Seq data from the 55 serous histology tumor samples. Model-based clustering was conducted using unsupervised clustering under the assumption that the “true” number of clusters were unknown in *mclust*[8]. Similarly to the simulation study, only the top 100 MAD genes were utilized during clustering. In this exploration, the Blom, Log, and VST transformations detected fewer clusters in comparison to data where no transformation was applied (Fig 4). Each data transformations identified different numbers of clusters ranging from 1, or no clusters, to 5 clusters.

## Discussion

For RNA-Seq data there have been few studies that have examined the clustering performance of model-based clustering in combination with data transformations. Hence, there is little guidance for researchers as to which data transformations should be used for RNA-Seq data when conducting model-based clustering, which tend to be based on Gaussian framework. Although clustering analyses are exploratory in nature, they can provide valuable information regarding the relationship between genes or samples. In order to provide this information, it is important to have accurate and efficient statistical methods. In light of the minimal information and studies currently available, we conducted and compared the results of an extensive simulation study to assess clustering method performance when data selection, data transformations, number of simulated clusters, and cluster sizes were varied. A strength of our study, over previously published studies, is the care at which the data was simulated using parameter estimates from a RNA-Seq study on ovarian cancer patients seen at the Mayo Clinic. Additionally, to combat biasing our performance results, datasets were simulated to represent four parent categories that considered the way in which the data were selected and the size of the clusters.

In terms of skewness, we determined that all transformations assessed made the data “more normal”. Specifically, the Blom transformation on average obtained the most normal data according to the mean skewness values (Table 1). The data transformations; however, did not provide any benefit in handling tail behavior as denoted by mean kurtosis values. In general, model-based clustering produced higher quality of clusters with more accuracy similar to findings in previous studies [7, 16, 34]. Since the primary model used in model-based cluster (*mclust*) is the Gaussian mixture model, it is reasonable that transforming data to look more normal would be highly beneficial concerning performance. Our results highly favor the use of the Log, base 2, transformation when it comes to conducting model-based clustering analysis of RNA-Seq data. VST also proved to be more favorable than the Blom and naïve data transformations.

This study has provided evidence that there is room to advance model-based clustering to utilize a mixture of discrete distributions, preferably a mixture of negative binomial distributions to capture the over-dispersion that is present in RNA-Seq data. In doing so, there would be no need for the additional step of transforming the data before conducting clustering analysis. Additionally, this study could be expanded, as only a subset of data transformations were assessed. It is highly likely that there is not one best data transformation. This also applies to expanding the simulation study to include other clustering methods, beyond model-based clustering, to determine how data transformations impact clustering performance. Specifically, it may be beneficial to explore nonparametric cluster methods to avoid being tied to model-based assumptions. Furthermore, this study serves to show that sample-based clustering has the ability to identify potential subtypes, and it aligns with other findings in current literature where varying numbers of subtype have been observed [4–6].

In conclusion, we found that RNA-Seq data requires caution when conducting clustering analyses. This is supported by our efforts to improve the performance of clustering methods through data transformations and common methods used to determine the number of clusters in a dataset. Additionally, the simulation study has revealed some of the challenges and difficulties that still remain for completing clustering analysis in RNA-Seq data particularly in meeting the assumption of normality.

## Supporting information

S1 Table Raw data for top 100 MAD genes. Raw count values for the top 100 MAD genes with the 100 genes by 55 samples contained in the matrices rows by columns, respectively.
(XLSX)

S2 Table Raw data for 100 random genes. Raw count values for 100 random genes with the 100 genes by 55 samples contained in the matrices rows by columns, respectively.
(XLSX)
